# Investigating the effect of intelligent assistance systems on motivational work characteristics in assembly

**DOI:** 10.1007/s10845-023-02086-4

**Published:** 2023-02-22

**Authors:** Marvin Walczok, Tanja Bipp

**Affiliations:** Department of Psychology, Hauptstraße 47-51, 69117 Heidelberg, Germany

**Keywords:** Intelligent assistance systems, Work design, Motivational work characteristics, Voluntary use, Assembly

## Abstract

*Intelligent assistance systems* (IAS) are designed to counteract rising cognitive demands caused by increasingly individualized manufacturing processes in assembly. How IAS affect *work characteristics* which are crucial for promoting work motivation of employees is yet unclear. Based on the *cyber-physical systems transformation framework*, the *model of routine-biased technological change*, and a comprehensive model of work design, we expected in- and decreases in motivational work characteristics (MWC) when working with IAS. Furthermore, we posited a buffering effect of the option of voluntary use on decreasing knowledge characteristics. Applying an online case study with *experimental vignette methodology* (EVM) allowed us to identify effects of the IAS on MWC before it is widely implemented. 203 German and British blue-collar workers evaluated an assembly workplace according to three experimental conditions (work without IAS, work with IAS, work with voluntary use of IAS). We identified enhanced feedback from the job and information processing in work with IAS in contrast to a traditional assembly workplace but found no restrictions (or elevations) in terms of other task (i.e., autonomy) or knowledge characteristics (i.e., job complexity, problem solving, specialization, skill variety). Thus, our results indicate that the IAS improves some motivational work characteristics of the assembly workplace, although it misses the primary goal of cognitive relief. Our study highlights the need for work design theories that specify the effect of IAS on motivational work characteristics and the potential benefit of IAS in assembly of the future.

**Supplementary Information:**

The online version contains supplementary material available at 10.1007/s10845-023-02086-4.

Despite technological advancements in today’s workplaces like additive manufacturing (Zhang et al., 2017) or collaborative robots (Faccio et al., 2023), assembly processes carried out by humans will remain indispensable in the digital factory of the future (Pfeiffer, 2016). Assembly work even becomes increasingly complex due to highly variable, individualized manufacturing processes (Egger-Lampl et al., 2019; Faccio et al., 2023). Various *intelligent assistance systems* (IAS) have been developed to effectively support assembly workers in their mostly monotonous but concentration-demanding jobs with the intention to reduce cognitive workload, and increase quality (Egger-Lampl et al., 2019; Kang et al., 2016; Stockinger et al., 2021). Although the (cognitive) support of assembly workers by such systems is intended (Berkers et al., 2022), the research field lacks specific theories and causal evidence on how IAS shape *motivational work characteristics* (MWC) (Baethge-Kinsky, 2020; Egger-Lampl et al., 2019). These are crucial for promoting work motivation, job satisfaction, and performance (Parker et al., 2017). Such insights are needed, given that the distribution of IAS is accelerated by the Covid-19 pandemic (Krzywdzinski et al., 2022), and several scholars have outlined potential risks of the implementation of IAS, such as restricted autonomy, or systematic de-skilling of employees (e.g., Blumberg & Kauffeld, 2020; Dostert & Müller, 2020).

Therefore, the aim of the current case study is twofold. First, using *experimental vignette methodology* (EVM) (Aguinis & Bradley, 2014), we are – to our knowledge – the first to systematically examine the causal effects of an exemplary projection-based, cognitive-assistive IAS on MWC to identify positive and negative effects before the IAS is implemented in practice. Second, we investigate whether potential negative effects can be mitigated by the voluntary use of IAS. By this, our study provides important contributions to theory and practice. On the one hand, our study aims to contribute to theory development with regard to the role of technology for work design models by shedding light on motivational effects of IAS. Furthermore, by focusing on the consequences of IAS for employees, we stress the importance of the still neglected, subjective human factors (Faccio et al., 2023) in the implementation of IAS in assembly. By examining subjective factors, we essentially extend current research on IAS which mainly focuses on objective evaluation indicators (Keller et al., 2019; Lampen et al., 2019). On the other hand, with regard to practice, our EVM study design provides a valuable tool to test the effect of specific (future) technologies on MWC. Overall, our case study offers valuable practical recommendations for human-centered design and implementation of IAS to create motivating workplaces in assembly of the future.

To make our expectations and contribution clear, we first introduce intelligent assistance systems (IAS) and their intended function in supporting assembly workers. In detail, we then define motivational work characteristics (MWC) and review the existing literature and theoretical models how IAS might affect MWC. Subsequently, we postulate and explain our expectations on the effect of a projection-based, cognitive-assistive IAS on nine different MWC. Then, we present the used methods, in particular outlining the implemented experimental vignette methodology (EVM). We then present the results of our hypothesis testing, and finally discuss the implications of our findings for theory and practice.

## Theoretical background

### Intelligent assistance systems at work

Technical systems that support people in carrying out activities by taking in and processing information from the environment or by inputting information are defined as intelligent assistance systems (IAS) (Hinrichsen, 2020). IAS represents an umbrella term covering a wide range of technical systems from wearables, including smart watches or data glasses (Blumberg & Kauffeld, 2020), to exoskeletons. Accordingly, IAS can be differentiated in their level of support or demand (low, medium, high, variable), type of support (physical, sensory, cognitive), and objective (compensatory, maintenance, enhancement) (see Apt et al., 2018 for a comprehensive overview of types of IAS).

In the current study, we focus on stationary, cognitive-assistive IAS with a low level of assistive performance, which will play an increasingly crucial role in modern assembly in tomorrow's smart factory (Apt et al., 2018). Such IAS are supposed to support workers cognitively by, for example, providing context-sensitive in-situ projection, or presenting work instructions and assembly information to reduce training times, workers’ uncertainties, avoid incorrect assembly steps, and consequently increase work productivity (Apt et al., 2018). They should also support the inclusion of non-native speakers to the workforce, low-skilled people, and those with other deficits through visual, language-independent instructional materials (Apt et al., 2018; Mark et al., 2019).

The IAS in our study (see Jung et al., 2022 for further technical characteristics of the IAS) consists of a 3D camera and a projector mounted overhead above the assembly workstation and an industrial PC. Furthermore, eye-trackers are integrated that allow tracking eye movements of employees during task execution. The system architecture consists of an information system with a semantically structured knowledge database and a control system. The 3D camera captures image and depth data and thus the hand movements of the assembly workers. With the help of algorithms, the hands are recognized in different levels of detail, and videos are converted into time series of position data. Activities are derived from further analysis of the time series. Machine learning principles enable work processes to be autonomously taught to the system by having the system repeatedly record a previously unknown assembly process. Automatically generated instructional material is projected onto the work surface in-situ as augmented reality via the projector. Thus, the IAS intends to support and cognitively relieve assembly workers by instructing each assembly step. A picture of the investigated intelligent assistance system as well as a text description of the presented job, assembly workplace, and functions of the system can be found in the ESM on the Open Science Framework (OSF). We used this information to introduce participants to the hypothetical assembly workplace in our empirical study.

Although such systems are not only developed but already used in practice, current research on IAS largely neglects the effects of these systems on human factors (Egger-Lampl et al., 2019; Faccio et al., 2023). Comprehensive models of how IAS shape the work design in assembly are still missing. Suggestions about the impact of IAS on work outcomes stem from the related field of *information and communication technology* (ICT). For example, researchers have reported contradictory impacts of ICT at work, for example, on motivation (Baethge-Kinsky, 2020; Day et al., 2019; Parker & Grote, 2022; Wang et al., 2020; Waschull et al., 2020). On the one hand, ICTs have the potential to replace dull routine work with algorithms and automation, giving workers more time to complete creative and cognitively demanding tasks, thereby increasing skill variety and motivation (Parker & Grote, 2022). On the other hand, ICTs could lead to a systematic de-skilling of employees by increasing automation of cognitively demanding work (Blumberg & Kauffeld, 2020; Parker & Grote, 2022). In turn, these reductions in skills can lead to lower motivation and learning-related outcomes (Parker & Grote, 2022). Still, current research on IAS largely neglects the effects of these systems on human factors despite its growing importance (Egger-Lampl et al., 2019; Faccio et al., 2023).

### Motivational work characteristics

*Work design* is defined as “the study, creation, and modification of the composition, content, structure, and environment within which jobs and roles are enacted” (Morgeson & Humphrey, 2008, p. 47), and has been shown to have a substantial impact on important outcome variables at work, such as work motivation, job satisfaction, or performance (Morgeson & Humphrey, 2006; Parker et al., 2017). Numerous theories and models have been suggested and tested in the last decades, to specify which work characteristics are linked to which work-related outcomes. For example, the *job characteristics model* (JCM) is one of the most influential theories in work design and motivation literature (Hackman & Oldham, 1976; Parker et al., 2017). It distinguishes five core job characteristics (job variety, job autonomy, job feedback, job significance, and job identity) which trigger three critical psychological states (experiencing meaning, feeling responsible for outcomes, and understanding the results of their efforts) which in turn have a positive impact on motivation (Hackman & Oldham, 1976; Parker, 2014).

Resulting from a review of different theories and empirical studies investigating work and job design, the *work design questionnaire* (WDQ) (Morgeson & Humphrey, 2006) is the most comprehensive and integrative instrument to capture work characteristics (Parker et al., 2017). Identifying key, yet distinct work characteristics, it includes aspects from established models such as the JCM (Hackman & Oldham, 1976), as well as further relevant identified work characteristics (Parker et al., 2017). The 21 work characteristics encompassed in the instrument have been shown to fall into four categories, namely *task, knowledge, social,* and *contextual work characteristics* (Morgeson & Humphrey, 2006). First, task characteristics capture the five JCM characteristics (Parker, 2014), which mainly refer to the execution of work and the “range and nature of tasks associated with a particular job” (Morgeson & Humphrey, 2006, p. 1323). In total, three facets of autonomy are separated (*work scheduling*, *decision-making,* and *work methods autonomy).* Further relevant task characteristics identified are *task variety, task significance, task identity,* and *feedback from the job*. Second, knowledge characteristics comprise knowledge, skills, and abilities needed for the execution of a job, and include *job complexity, problem solving, information processing, skill variety,* and *specialization* (Morgeson & Humphrey, 2006)*.* Because of their positive relation with motivation, task and knowledge characteristics are also referred to as motivational work characteristics (Morgeson & Humphrey, 2006; Parker, 2014; Stegmann et al., 2010). Providing evidence for their relevance, Humphrey et al. (2007) showed in their meta-analysis that these motivational characteristics together can explain up to 34% of the variance in job performance, job satisfaction, internal work motivation, or organizational commitment. Third, the WDQ also covers social and contextual characteristics, which refer to the social environment in which the work is embedded and the physical nature of the work environment, respectively (Morgeson & Humphrey, 2006). In our current study, we focus on the motivational work characteristics because of their importance for work motivation, and their fundamental changes due to technological changes at work (Parker & Grote, 2022; Wang et al., 2020).

### Altered work design characteristics due to the implementation of IAS

Although the WDQ provides an organizing framework of work characteristics and has stimulated research about the effects of motivational, social, and context characteristics at work, specific theories and empirical evidence on how work characteristics are altered by the introduction of technology in the workplace are missing. This could be because the effect of technology on work characteristics depends, among other things, on the specific type and design of technology (Parker & Grote, 2022). Gagné et al. (2022) state that digital technologies have the potential to in- and decrease motivational work characteristics, as “there is no deterministic relationship between technology and work design; instead the effect of new technology on work design, and hence motivation, depends on various moderating factors” (p. 6), such as the employees’ skills or organizational implementation factors. Therefore, the existing literature on the effects on work design appears to be too general for a generalization to stationary IAS in assembly. For example, wearables enable time- and location-flexible working and thus increase an employee’s autonomy (Parker & Grote, 2022). However, in contrast, such effects seem not easily transferable to stationary IAS, as they do not support such work arrangements. This highlights the importance of technology-specific studies; in our case, this is a stationary, cognitive-assistive IAS.

On a general level, different theoretical models or frameworks stemming from various fields (i.,e. industrial engineering, or economics) might help to explain some of the motivational effects on task and knowledge characteristics when introducing IAS at work. First, the *CPS transformation framework* by Waschull et al. (2020) is based on the WDQ (Morgeson & Humphrey, 2006). It postulates that job autonomy, job complexity, and skill variety in industrial production are affected by the introduction of cyber-physical systems, for example, IAS (Drossel et al., 2018). In detail, the authors expect an increase in job complexity as simple tasks will be eliminated by increasing automated information collection and analysis, whereas complex tasks will continue to exist. This framework also postulates significant increases in skill variety, especially in occupations that require high levels of information processing. For low-skilled and middle-skilled jobs like jobs in assembly (Maxwell, 2006), the authors suggest reductions in autonomy, as technologies might limit decision-making freedom, clock and standardize work (Waschull et al., 2020). Although this framework allows postulating specific effects of the introduction of IAS on task and knowledge characteristics, so far empirical evidence for these postulations in a comprehensive work design model is—to our knowledge—still missing.

Second, Autor et al. (2003) developed the *model of routine-biased technological change*, which postulates a polarization of jobs in the form of an increasing number of low-skilled and high-skilled jobs as the number of middle-skilled jobs decreases due to the introduction of digital technologies. It differentiates between manual and cognitive as well as routine tasks—tasks that “can be performed by machines according to explicitly programmed rules” (Autor et al., 2003, p. 1283)—and non-routine tasks. While increasing automation through new digital technologies is expected for manual and cognitive routine tasks, the model posits strong complementarities for non-routine cognitive tasks as well as limited opportunities for substitution or complementarities for non-routine manual tasks (Autor et al., 2003). Based on this model, Mlekus (2021) empirically showed that the relationship between digitization level in the workplace and competency requirements is indeed moderated by the context (stronger focus on nonroutine manual vs. nonroutine cognitive tasks). Whereas she found a positive correlation between digitalization level and competence requirements in the domain of production (stronger focus on nonroutine cognitive tasks), a negative correlation between digitization level and competence requirements in the domain of logistics was evident (stronger focus on nonroutine manual tasks) (Mlekus, 2021). Modern assembly is characterized by nonroutine cognitive and nonroutine manual tasks due to the highly frequent switching of assembly processes due to highly variable, individualized manufacturing processes (Egger-Lampl et al., 2019; Faccio et al., 2023). The implementation of IAS in assembly should therefore not only increase the digitalization level in the workplace but also shift the focus to nonroutine manual tasks since employees are cognitively supported by the use of IAS so that nonroutine cognitive tasks play a subordinate role. Consequently, the model of routine-biased technological change suggests a reduction of knowledge characteristics while working with IAS, while it is unclear yet which particular aspects are indeed positively or negatively affected.

### Overview of present research and hypotheses

In sum, in the context of IAS at work, there are mainly theoretical considerations about the extent to which motivational work characteristics will be transformed instead of empirical evidence. Existing findings regarding IAS apply to wearables such as smart watches or smart glasses (Blumberg & Kauffeld, 2020; Paruzel et al., 2020). Hence, we aim to provide the first systematic investigation of the effects of stationary IAS on motivational work characteristics within a comprehensive framework of work design. Based on the CPS transformation framework and the model of routine-biased technological change framework, we suggest that IAS not only affect motivational work characteristics positively but also carry the risk of detrimental effects (see Table 1 for an overview of the closely related empirical work on IAS and motivational work characteristics). Furthermore, we propose that the voluntary use of IAS can mitigate negative effects on knowledge characteristics.

**Table 1:** Closely related empirical work on intelligent assistance systems (IAS) and motivational work characteristics (MWC)

Authors (Year)	Method and sample	Relevant findings with regard to MWC
Blumberg & Kauffeld (2020)	Interview study with 76 German experts from science, politics, and industrial practice	Experts indicate risks of restricted autonomy and systematic de-skilling of employees when using smart watches or data glasses in industrial practice
Berkers et al. (2022)	Interview study with 24 Dutch employees and managers from six logistic organizations	Warehouse workers cite the fear of restricted autonomy by implementation of robots in logistic warehouses
Lampen et al. (2019)	Experiment with 24 German students and research engineers realizing a within-subject design (3 instructions: Pictorial paper-based, 3D in-situ-visual cues, human simulation approach)	Cognitive workload in learning new tasks is significantly reduced by using the 3D in-situ-visual cues instruction compared to the pictorial paper-based instruction
Paruzel et al. (2020)	Cross-sectional survey with 14 German employees in manufacturing company on expectations and fears of the implementation of smart glasses	The employees expect potential positive and negative effects of smart glasses on work characteristics (e.g., increased and decreased job complexity)

First, with regard to the task characteristics included in the WDQ, we expected distinct effects of the introduction of IAS on autonomy and feedback. Taking a closer look at the three facets of autonomy, work scheduling autonomy refers to “the extent to which a job allows freedom, independence, and discretion to schedule work” (Morgeson & Humphrey, 2006, p. 1323), whereas decision-making autonomy reflects the degree to which a job allows incumbents to make decisions on their own. The facet of work methods autonomy is defined as the extent to which a job allows workers to choose methods to perform their tasks themselves (Morgeson & Humphrey, 2006). IAS are introduced with the overall goal to standardize work by specifying and providing information about subsequent work steps, detailed sequences of individual assembly steps, instruments or tools to be used for specific tasks, as well as solutions to problems at each point in the execution of activities (Blumberg & Kauffeld, 2020; Dostert & Müller, 2020; Waschull et al., 2020). Therefore, their introduction at the workplace suggests reductions in all three autonomy facets. Such an expectation is supported by the qualitative studies (cf. Table 1) by Blumberg and Kauffeld (2020) and Berkers et al. (2022). Several interview participants highlighted the decline of autonomy as a central risk of the implementation of data glasses and tablets as IAS at work (Blumberg & Kauffeld, 2020), and the implementation of robots in logistics (Berkers et al., 2022). However, causal evidence for such an effect for IAS in assembly is so far missing. Furthermore, specifying the effects of the implementation of IAS on all three different autonomy facets seems necessary, as these facets are differently related to work outcomes (Humphrey et al., 2007).

#### Hypothesis 1

(a) Work scheduling autonomy (b) Decision-making autonomy (c) Work methods autonomy is significantly lower in work with IAS than in work without IAS.

With regard to feedback from the job, defined as “the degree to which the job provides direct and clear information about the effectiveness of task performance” (Morgeson & Humphrey, 2006, p. 1323), significant increases can be expected through the implementation of IAS, as they provide feedback on every assembly step and can be used specifically for learning a specific task or job (Apt et al., 2018).

#### Hypothesis 2

Feedback from job is significantly higher in work with IAS than in work without IAS.

Concerning further task characteristics included in the WDQ, we expect neither positive nor negative effects of IAS on task variety, task significance, and task identity, since the individual work steps and thus the work as a whole remain largely unchanged. The individual work steps are merely assisted by the IAS. This is supported by findings based on qualitative interviews with production workers from Baethge-Kinsky (2020), who reported that work with IAS still has the same features (high monotony, routine tasks, and few opportunities for technically demanding activities) as work without IAS unless the work is extended through work design interventions.

Second, we expected all five knowledge characteristics to be affected by the introduction of IAS. First, job complexity describes how complex and difficult the tasks that the job holder has to perform are (Morgeson & Humphrey, 2006). The two general frameworks do not convey in this regard: While the CPS transformation framework (Waschull et al., 2020) postulates significant increases, reductions can be expected based on the routine-biased technological change model (Autor et al., 2003). However, as the IAS intends to support employees cognitively, it can be expected that job complexity decreases as it provides precise instructions for each assembly step (Apt et al., 2018). Furthermore, such systems have been associated with the risk of long-term systematic de-skilling of employees by taking over the mental work (Baethge-Kinsky, 2020; Blumberg & Kauffeld, 2020; Parker & Grote, 2022). Also, Lampen et al. (2019) showed that the cognitive workload learning a new task can be significantly reduced by an IAS compared to a paper instruction. Therefore, we expected that job complexity will be reduced while working with an IAS.

#### Hypothesis 3

Job complexity is significantly lower in work with IAS than in work without IAS.

Second, problem solving describes “the degree to which a job requires unique ideas or solutions and reflects the more active cognitive processing requirements of a job” (Morgeson & Humphrey, 2006, p. 1323). Since IAS uses, for example, context-sensitive in-situ projections to instruct workers on subsequent assembly steps and identify incorrect activities as mistakes, problem solving is taken over by IAS so that workers no longer have to solve problems on their own (Dostert & Müller, 2020). Due to this, knowledge, skills, and abilities regarding problem solving can decrease in the long term, potentially leading to a systematic de-skilling of workers (Autor et al., 2003), a potential risk that was also mentioned by some interview participants in the study by Blumberg and Kauffeld (2020).

#### Hypothesis 4

Problem solving is significantly lower in work with IAS than in work without IAS.

Third, information processing refers to “the degree to which a job requires attending to and processing data or other information” (Morgeson & Humphrey, 2006, p. 1323). On the one hand, the IAS as another source of information in addition to the traditional workplace provides information that has to be monitored and processed by the employees, so the demands on information processing could increase through the introduction of IAS. On the other hand, there is also the possibility of a decrease in information processing because IAS aim to support the job incumbents cognitively and could take over the cognitive work entirely ( Baethge-Kinsky, 2020; Blumberg & Kauffeld, 2020). This also seems plausible considering the strong intercorrelation of information processing with other knowledge characteristics, in terms of problem solving and complexity (Morgeson & Humphrey, 2006; Stegmann et al., 2010). Due to the plausibility of the two directions and the lack of empirical research regarding changes in information processing through the implementation of IAS, we suggest competing hypotheses for this work characteristic.

#### Hypothesis 5

Information processing is significantly (a) lower (b) higher in work with IAS than in work without IAS.

Fourth, skill variety reflects the necessary amount of skills that are required for the completion of the work (Morgeson & Humphrey, 2006). Similar to information processing, significant reductions or significant increases in the skill variety through the introduction of IAS seem plausible. On the one hand, IAS are specifically used to train non-native speakers and low-skilled persons and integrate them into the labor market (Apt et al., 2018; Mark et al., 2019), so that the skill variety could decrease significantly by taking over the cognitive work. This would again result in the risk of systematic de-skilling or general downgrading (Autor et al., 2003; Baethge-Kinsky, 2020; Blumberg & Kauffeld, 2020). On the other hand, in addition to the traditionally needed requirements, digital competencies are required for successful interaction with the IAS (Waschull et al., 2020), which will play an ever-increasing role in the context of digitalization, smart factory, and Industry 4.0 (Oberländer et al., 2020). This may even result in general upskilling (Baethge-Kinsky, 2020).

#### Hypothesis 6

Skill variety is significantly (a) lower (b) higher in work with IAS than in work without IAS.

Fifth, specialization refers to “the extent to which a job involves performing specialized tasks or possessing specialized knowledge and skill” (Morgeson & Humphrey, 2006, p. 1324). In terms of specialization, the introduction of IAS can be expected to lead to a deterioration, as, for example, the context-sensitive in-situ instructions allow non-native speakers and low-skilled individuals to be trained more quickly, thus requiring fewer specific skills and abilities (Apt et al., 2018), which could result in a systematic de-skilling of employees (Autor et al., 2003; Baethge-Kinsky, 2020; Blumberg & Kauffeld, 2020).

#### Hypothesis 7

Specialization is significantly lower in work with IAS than in work without IAS.

Besides investigating the effects of the introduction of IAS in assembly on core work characteristics, we were interested if the potential negative effects of using such a system (cf. our expected effects in terms of lowered knowledge characteristics outlined above), can be buffered in practice. Using the system voluntarily-allowing employees to use the system or not-could counteract systematic de-skilling of workers based on the model of routine-biased technology change (Autor et al., 2003). By preventing over-support and excessive takeover of cognitive work, modern assembly continues to include not only nonroutine manual but also nonroutine cognitive tasks for which the model expects strong complementarities in skills (Autor et al., 2003) which has been empirically supported (Mlekus, 2021). Therefore, we suggest, that using the system voluntarily could buffer potential negative effects of IAS on knowledge characteristics by preventing a shift in focus to nonroutine tasks.

#### Hypothesis 8

(a) Job complexity (b) problem solving (c) information processing (d) skill variety (e) specialization is significantly higher in work with voluntary use of IAS than in work with IAS.

## Method

In our experiment, we investigated the causal impact of a stationary, cognitive-assistive IAS on task and knowledge characteristics and the role of the voluntary use of IAS concerning knowledge characteristics. For this, we manipulated an assembly workstation using EVM in an online experiment realizing three experimental conditions (work without IAS vs. work with IAS vs. work with voluntary use of IAS).

### Open science

We pre-registered all procedures and hypotheses before data collection (https://osf.io/352zd/?view_only=5ff4cf3e692d44edbb0843418bb9bc26). The electronic supplementary material (ESM) including the presented hypothetical assembly workstation, information on the presented IAS, and additional analyses can be found in the OSF.

### Experimental design and procedure

We manipulated the assembly workstation in an online study using a between-subjects design based on vignettes with three experimental conditions: work without IAS vs. work with IAS vs. work with voluntary use of IAS. Since the implementation of IAS in assembly is still in its infancy (Bortolini et al., 2021) which complicates the experimental investigation of the effect of such systems with samples in organizational practice, we chose an EVM study design. Such a study design is associated with several advantages. First, it allows the investigation of causal effects (high internal validity) using realistic (future) scenarios, maximizing the generalizability of experimental results (high external validity) (Aguinis & Bradley, 2014). Hence, our study enables the much-needed identification of potential opportunities and risks of such systems before they are implemented. Second, and in contrast to, for example, an investigation of the changes in work characteristics before and after the implementation of an IAS in one organization, the EVM allowed us to investigate employees from diverse organizations, thus increasing generalizability of our results. And implementing the exact same system in various organizations and investigating that across them seems not feasible, as different organizations would need to adapt the IAS to the specific needs of their particular assembly processes, which would systematically bias the results. In this way, the EVM study counteracts assembly process- and organization-specific effects, such as an organization’s technology-averse tendency. Third, the EVM study design allows the pure investigation of the effect of the IAS on MWC without the impact of additional interferences, such as time delays or incorrect modeling which could occur in occupational practice (Tao et al., 2022; Xu et al., 2021).

**Fig. 1:**
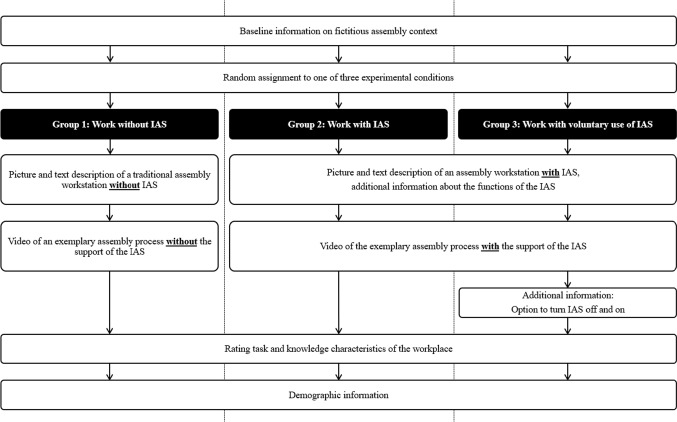
Flow chart of the study with three experimental groups (Group 1: Work without IAS, Group 2: Work with IAS, Group 3: Work with voluntary use of IAS). IAS Intelligent assistance system

In our realization of the EVM in an online study, we followed the best practice recommendations for designing and implementing such studies by Aguinis and Bradley (2014) and used baseline information, and combined text, picture and video material to maximize the level of immersion. Figure 1 includes a flow chart depicting procedure of the experiment. In the beginning, we asked all participants who agreed to participate to imagine a hypothetical work situation as an assembly worker in a medium-sized assembly company. Therefore, all participants read a three-sentence long baseline information on the situational context. Afterward, participants were randomly assigned to one of the three experimental conditions.

In the first condition, work without IAS, participants were given a page of text description of a traditional assembly workstation (including a picture of the workspace) without IAS as well as an approximately 1-min long video of an exemplary assembly process without the support of the IAS.

In the second condition, work with IAS, and the third condition, work with voluntary use of IAS, participants were given a page of text description of an assembly workstation with IAS (again including a picture of the workspace) and additional information about the functions of the IAS. Also, the participants watched an approximately 1-min long video of the exemplary assembly process with the support of the IAS. Conditions 2 and 3 differed only in that the participants in Condition 3 received the information that they could turn the IAS off and on at any time on a separate page. The IAS presented in the vignette is a projection-based, cognitive-assistive system that guides workers through each assembly step using short instructional videos projected onto the work surface. If the work steps are performed correctly, the projected video will light up green; if they are performed incorrectly, the video will light up red. The progress of work processes is displayed in the form of a progress bar above the videos with the assembly steps on the work surface. Alternative workflows and assembly sequences can be observed and taught automatically using machine learning. The basis of these functions lies in the recording of the hand and eye movements of the employees with the help of a 3D camera and three eye trackers (Jung et al., 2022). A translation of the description of the hypothetical workplace with IAS can be found in ESM (Online Appendix A). The video of the presented assembly process with(out) the support of the IAS can be found in the pre-registration in the Open Science Framework.

To ensure careful reading of the text description and observation of the assembly process, we included a time lag of 1 min each before one could continue with the study in all conditions. Subsequently, participants rated the workplace according to their condition regarding task and knowledge characteristics in randomized order and finally answered some demographic information (age, sex, and prior experiences with IAS) about themselves.

### Participants

For recruiting participants, we realized two approaches. First, we invited German blue-collar workers from our personal and professional networks from diverse fields via email and social media to participate in an online experiment. Blue-collar workers perform jobs relatively similar to assembly work, so they are able to adequately assess the work situation presented. The prerequisite for participation was a current job or prior work-related experience in the areas of manufacturing, assembly, or craftsmanship. We offered them a summary of the study results and an opportunity to enter a drawing for vouchers (5 × 20 Euro) for every 50 participants as incentives for participating. Within the collection period from October 2021 to April 2022, 1,041 people clicked on the link to the online experiment. 135 participants completed the survey (response rate of approximately 13%). We excluded six participants who failed the attention check (“Please tick answer option 1 ‘do not agree at all’ in this row”), five participants who had technical problems, and another 23 who classified their job as an office job and had no prior experience working in manufacturing, assembly, or craftsmanship from the analysis. This led to 101 German participants.

Second, we collected additional data from British blue-collar workers with an English version of our experiment in April 2022 using Prolific. We paid 2.20 GBP for participating. Of the 147 people who clicked on the link to the study, 127 participants completed the experiment (response rate of approximately 86%). We excluded two participants who failed one of two built-in attention checks. We further excluded 23 participants who reported that they were currently working in an office job or were students and did not have any prior work experiences in manufacturing, assembly, or crafting, resulting in 102 British participants.

Given the two different data collection efforts, we checked for potential differences in demographic variables. We did not find significant differences in age, *t*(201) = − .826, *p* = .410, *d* = − .116, sex, χ^2^(2) = 0.334, *p* = .846, φ = .041, or prior experience with working with IAS, χ^2^(1) = 1.012, *p* = .314, φ = − .071, between the German and British participants (ESM, Table 2). Furthermore, a chi-square difference test revealed no disproportionate allocation of the German or English subsample to one of the three experimental conditions, χ^2^(1) = 1.159, *p* = .560, Cramér’s V = 0.076 (ESM, Table 2).

Therefore, we based our analysis on the combined data, resulting in a sample of *N* = 203. Ages ranged from 19 to 75 (*M* = 37.54, *SD* = 12.01). The majority of the participants were male (70.4%) and had no prior experience with IAS (87%). Among those 13% of participants with prior experience with IAS, virtual reality (*n* = 10), pick-by-light (*n* = 6), and augmented reality (*n* = 3) were the most common. The low number of participants with prior experience with working with IAS in our sample reflects the low use and distribution of IAS in German and British production companies, highlighting the importance of EVM study designs.

### Manipulation checks

First, to ensure the successful manipulation of the assembly workstation, we asked participants in all three experimental conditions to rate *equipment use* in the hypothetical workplace immediately after reading the text description and watching the short video of an exemplary assembly process. For this, we used three items of a validated German version (Stegmann et al., 2010) (α = .76) or the validated English version of the WDQ (Morgeson & Humphrey, 2006), with a five-point Likert scale ranging from *strongly disagree* (1) to *strongly agree* (5). We conducted a Helmert contrast to test whether the work without IAS differs significantly from the other two conditions (work with IAS and work with voluntary use of IAS). Equipment use was rated significantly lower in the condition of work without IAS (*M* = 2.00, *SD* = 0.92) than in the other two conditions (work with IAS: *M* = 2.29, *SD* = 0.97; work with voluntary use of IAS: *M* = 2.26, *SD* = 0.86), *F*(2, 199) = 3.29, *p* = .039, η_*p*_^2^ = .032, *C* = − .314, *p* = .014, indicating that our experimental manipulation of the assembly workstation was successful.

Second, to ensure the successful manipulation of voluntary use of the IAS, we asked participants to rate work methods autonomy (α = .91) from the WDQ (Morgeson & Humphrey, 2006) to check our experimental manipulation of the voluntary use of IAS between conditions work with IAS and work with voluntary use of IAS. We used three validated items of the German version (Stegmann et al., 2010) or the English version (Morgeson & Humphrey, 2006) with the same five-point Likert scale as equipment use. A t-test indicated that the work with voluntary use of IAS condition (*M* = 1.92, *SD* = 0.97) did not differ significantly from the work with IAS condition (*M* = 1.62, *SD* = 0.87) in terms of work methods autonomy, *t*(135) = − 1.468, *p* = .144, *d* = − .251, resulting in a failed manipulation of voluntary use. Hence, we combined the work with IAS and work with voluntary use of IAS into the work with IAS condition (see ESM, Table 4 for ANOVA results of all three experimental conditions). This precluded us from testing H8.

### Measures

#### Task and knowledge characteristics

We measured the four task characteristics and the five knowledge characteristics based on the original English version of the WDQ (Morgeson & Humphrey, 2006) and its validated German version (Stegmann et al., 2010). In total, participants rated 32 items on a five-point Likert scale with the range from *strongly disagree* (1) to *strongly agree* (5). To cover the hypothetical job situation (and not the current job participants hold), we slightly altered the item wording of some German items (items including “my job” were changed to “the job”). This was not necessary for the English version by Morgeson and Humphrey (2006) where “the job” was already used. In detail, we measured work scheduling autonomy (“The job allows me to plan how I do my work”, α = .86), decision-making autonomy (“The job allows me to make a lot of decisions on my own”, α = .89), work methods autonomy (“The job allows me to make decisions about what methods I use to complete my work”, α = .91), and feedback from job (“The job itself provides feedback on my performance”, α = .86) with three items each. To capture the knowledge characteristics job complexity (“The tasks on the job are simple and uncomplicated”, α = .86), problem solving (“The job requires to be creative”, α = .84), information processing (“The job requires me to monitor a great deal of information”, α = .91), skill variety (“The job requires a variety of skills”, α = .92), and specialization (“The job requires a depth of knowledge and expertise”, α = .91), we used four items each. The internal consistency reliabilities (Cronbach’s α) ranged from .79 to .92. Table 3 in the ESM also contains reliabilities of the German and English versions separately.^1^

## Results

**Fig. 2:**
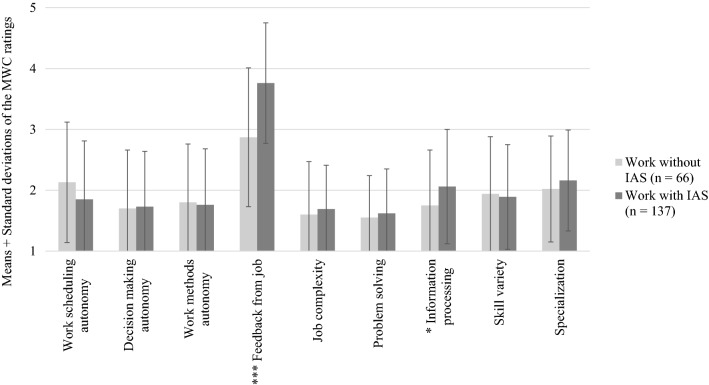
Ratings of motivational work characteristics in work without IAS and work with IAS. IAS Intelligent assistance system. MWC Motivational work characteristics. *p < .05. ***p  < .001

Table 2 shows the descriptive statistics of the two experimental conditions work without IAS and work with IAS. The ratings of all motivational work characteristics are low across all conditions. A one-way MANOVA showed a significant difference between the two working conditions without and with IAS for all nine motivational work characteristics under investigation,^2^*F*(9, 193) = 6.441, *p* < .001, η_*p*_^2^ = .231, Wilk’s Λ = .769. Subsequently, we conducted t-tests for every motivational work characteristic separately according to our hypotheses (Table 2 and Fig. 2). We did not find a significant difference between work without IAS and work with IAS in work scheduling autonomy, *t*(201) = 1.909, *p* = .058, *d* = .286, decision-making autonomy, *t*(201) = − 0.149, *p* = .882, *d* = − .022, and work methods autonomy, *t*(201) = 0.330, *p* = .742, *d* = .049, thereby rejecting H1a-c. Feedback from job was significantly higher in work with IAS than in work without IAS, *t*(201) = − 5.701, *p* < .001, *d* = − .854, supporting H2. There was no significant difference between work without IAS and work with IAS in job complexity, *t*(201) =  − 0.790, *p* = .430, *d* = − .118, and problem solving, *t*(201) =  − 0.728, *p* = .467, *d* = − .109. Thus, we rejected H3 and H4. Information processing was significantly higher in work with IAS than in work without IAS, *t*(201) = − 2.205, *p* = .029, *d* = − .330, supporting H5b. Finally, the two working conditions did not differ in skill variety, *t*(201) = 0.369, *p* = .713, *d* = .055, and specialization, *t*(201) = − 1.077, *p* = .283, *d* = − .161. Hence, we rejected H6a, H6b, and H7.

**Table 2:** Cell means and standard deviation of motivational work characteristics in work without IAS and work with IAS

	Work without IAS		Work with IAS		t-test	Hypothesis
	*n* = 66		*n* = 137			
	*M*	*SD*	*M*	*SD*		
Work scheduling autonomy	2.13	0.99	1.85	0.96	*t*(201) = 1.909, *p* = .058, *d* = .286	1a
Decision-making autonomy	1.70	0.96	1.73	0.91	*t*(201) = − 0.149, *p* = .882, *d* = − .022	1b
Work methods autonomy	1.80	0.96	1.76	0.92	*t*(201) = 0.330, *p* = .742, *d* = .049	1c
Feedback from job	2.87	1.14	3.76	0.99	*t*(201) = − 5.701, *p* < .001, *d* = − .854***	2
Job complexity	1.60	0.87	1.69	0.72	*t*(201) = − 0.790, *p* = .430, *d* = − .118	3
Problem solving	1.55	0.69	1.62	0.73	*t*(201) = -0.728, *p* = .467, *d* = − .109	4
Information processing	1.75	0.91	2.06	0.94	*t*(201) = − 2.205, *p* = .029, *d* = − .330*	5a/b
Skill variety	1.94	0.94	1.89	0.86	*t*(201) = 0.369, *p* = .713, *d* = .055	6a/b
Specialization	2.02	0.87	2.16	0.83	*t*(201) = − 1.077, *p* = .283, *d* = − .161	7

*IAS* Intelligent assistance system

*p < .05. ***p < .001

## Discussion

As prior research on IAS largely neglected human factors (Egger-Lampl et al., 2019; Faccio et al., 2023), our case study contributes to a human-centered design and integration of advanced technologies in assembly in two important ways. First, we were able to provide causal evidence for an increase in MWC feedback from job and information processing due to the implementation of a cognitive-assistive IAS in assembly. Whereas the effect of the IAS on information processing was rather weak, we found a strong effect on feedback from the job. As our results are based on a rather big sample stemming from two European countries highlights that such effects might not be bound to specific countries. Second, unfortunately, we were not able to provide insights into a potential buffering effect of voluntary use, as our manipulation of voluntary use failed. However, the results of our case study in this regard are also promising, since we were not able to identify any essential decrease in knowledge characteristics, indicating no negative consequences of working with IAS that should alarm practice. In sum, our results provide important insights into the work design and IAS literature as well as practical implications for the potential benefits of IAS in assembly.

### Theoretical implications

Although the CPS transformation framework (Waschull et al., 2020) postulates restrictions in autonomy in low- and middle-skilled jobs by clocking and standardizing work, we did not find reduced autonomy due to the IAS as suggested in qualitative studies (Berkers et al., 2022; Blumberg & Kauffeld, 2020). This applies to the three autonomy facets distinguished in the WDQ, extending the current state of limited research in which autonomy is considered a global construct without considering relevant autonomy facets (Blumberg & Kauffeld, 2020). The fact that the IAS does not seem to decrease perceived autonomy is probably attributable to the already highly standardized traditional assembly work. Additionally, in our description of the system, we also indicated that the system can adapt to alternative work processes using machine learning. For example, in the case of work scheduling autonomy, this could mean that alternative sequences in the assembly process preferred by employees may counteract decreases. It is not surprising that IAS can be used to foster immediate feedback from the job which highlights their importance in phases in which frequent and immediate feedback is needed. The model of routine-biased technological change (Autor et al., 2003) posits a reduction in knowledge characteristics due to new technologies, resulting in a de-skilling of employees in assembly by automating manual and cognitive routine tasks. However, our results indicate that the IAS will not establish a lower level of requirements on the assembly workers, since problem solving, skill variety, and specialization seem to be unaffected by the implementation of the IAS. The results also do not indicate a higher skill variety, for example, with an associated need for digital competencies (Oberländer et al., 2020) when working with IAS. These knowledge characteristics appear to be more dependent on the underlying assembly process than on the IAS. Given that job complexity is not lowered by the IAS investigated here either, it appears that the system is failing to achieve its primary goal of cognitive relief of employees (Egger-Lampl et al., 2019). The introduction of IAS even requires assembly workers to pay attention to an additional source of information and thus to process more information which is generally associated with positive work outcomes (Stegmann et al., 2010). Therefore, our results suggest an improvement regarding work motivation in the digitized assembly workplace, although the IAS does not provide the cognitive relief for which such systems were primarily developed. Overall, our results stress the importance of subjective human factors (Faccio et al., 2023) when implementing IAS in assembly.

The fact that the majority of our hypotheses had to be rejected highlights the need for theories targeting the specific impact of innovative technologies, such as IAS, on work characteristics. The CPS transformation framework and the model of routine-biased technological change did not provide either a suitable basis for predicting the influence of cognitive-assistive IAS on work design, as they do not take into account the specific technical design features (Gagné et al., 2022). However, by applying a case study on an exemplary IAS and its effect on motivational work characteristics, we contribute to the development of such theories.

### Practical implications

Although prior qualitative studies (e.g., Berkers et al., 2022; Blumberg & Kauffeld, 2020) mainly emphasized the negative sides of IAS, namely the risks of reduced autonomy and de-skilling of employees, our results indicate no such effects and even an improvement of certain aspects of the digitized assembly workplace. This could prove beneficial for reducing employees’ resistance to change and support the introduction of such systems in practice. Assembly workers can benefit in terms of work-related outcomes, like motivation and job satisfaction, by using an IAS primarily through these two work design aspects. First, they profit from the strongly enhanced feedback from the job which is positively associated with motivation (Humphrey et al., 2007). The enhanced feedback implies that especially in the learning and training phases, assembly workers can benefit from the use of IAS (Doolani et al., 2020). Providing immediate feedback from the job by using IAS will also help in the long term if the work processes are characterized by individualized customer requests and the associated frequent product changes as in modern assembly. Second, assembly workers can benefit from increased information processing when working with the IAS compared to the traditional workplace which is positively associated with job satisfaction (Humphrey et al., 2007). Nevertheless, managers must be aware that the increased information processing may represent a double-edged sword. Since IAS also aim at the inclusion of low-skilled workers and workers with cognitive deficits (Apt et al., 2018; Mark et al., 2019), enhanced information processing could potentially lead to information overload. However, as we found a rather weak effect in terms of effect size, thus the additional source of information should not pose a great risk for employees without cognitive deficits.

On a more general level, the overall rather low ratings in MWC for both, the traditional and digitized workplace in our vignette emphasize the need for work design interventions in assembly, such as *task* or *job rotation* (Mlekus & Maier, 2021). Task rotation “refers to the alternation between tasks within a job that can require different skills and responsibilities but is not associated with a change to a different function or department” (Mlekus & Maier, 2021, p. 2), in this case, the alternation between assembly products. This task rotation is already an essential consequence of the individualized customer requests that characterize the digital factory of the future. Job rotation is defined as “a lateral transfer of employees within an organization without a change in salary or hierarchy” (Mlekus, 2021, p. 2) and can include rotations not only in departments but whole units (Mlekus & Maier, 2021). Applying job rotation on a larger scale will be more challenging than using task rotation in this assembly context. The implementation of our exemplary IAS as an innovative advanced technology could represent a first step in improving MWC of assembly workplaces.

### Limitations and future research

Although our case study provides first causal insights into alterations in MWC by the implementation of a stationary, cognitive-assistive IAS in assembly, some limitations need to be acknowledged. First, our results might be limited due to the rather low ratings in the MWC across all experimental conditions. That we did not find any negative effects of the IAS on MWC might therefore also be due to potential floor effects in the ratings, as the presented assembly process was highly simplified. The investigation of the effect of IAS with more demanding assembly processes could counteract such effects, particularly for expected effects on knowledge characteristics.

Second, our manipulation of the voluntary use of IAS failed. Participants in the work with IAS condition may have implicitly assumed that they could turn the system off and on at any time. However, we did not include explicit coercion in this condition to avoid artificially biasing the study results, as some studies show that coercion elicits negative responses from employees (Hausman & Johnston, 2010; Yılmaz & Kılıçoğlu, 2013). Again, the investigated exemplary assembly process may also have contributed to this missing effect, as cognitive relief from the IAS is not essential for a highly simplified assembly process. This is also evident from technology acceptance models (Feng et al., 2021), where the perceived usefulness of a system is a key predictor of the intention to use it. Consequently, individuals may have thought that they could simply ignore the system if it did not fulfill the desired goal of cognitive relief. This may also be a potential reason why we found no negative consequences of the system on MWC. If employees assume over-simplified work processes with restricted autonomy when working with IAS, they will not use the system.

Third, the use of an EVM study design allowed causal investigation of the effect of the IAS on MWC, taking into account specific design features, such as autonomous teaching of the system through machine learning, even before the system is implemented in practice. Nonetheless, participants were not asked to work with the presented IAS, thus results may be biased by their expectations of such systems. However, given the experimental design of our study, the random assignment to one of the three conditions prevented at least systematic distortions of particular expectations. Future studies should assess MWC before and after implementing IAS on the work floor to investigate the specific effect of the system under more realistic conditions, over a longer time period. In addition, the EVM study design prevented us from collecting other (objective) assessment factors, such as the task completion time, or error count, which have already been investigated in other studies (Keller et al., 2019; Lampen et al., 2019). In future studies, subjective factors, as in our study, should be supplemented with objective factors to provide an even more comprehensive picture of the effect of IAS on motivational and performance-related outcomes in terms of quality and quantity.

Fourth, we explicitly omitted sources of interference in the representations of the assembly workplace with IAS to investigate its pure effect on MWC. However, interferences, such as time delays or modeling errors (Tao et al., 2022; Xu et al., 2021), can be expected to occur in operational practice in assembly process planning (Qian et al., 2023) when working with IAS, which could alter the experiences of workers with such systems. Depending on whether assembly workers need to fix certain malfunctions on their own, knowledge characteristics could increase by using IAS, for example, because programming skills are needed to fix interferences but are not necessary for the majority of daily assembly processes. Thus, the role of such additional factors needs to be investigated in future studies.

Fifth, since the effects of advanced technologies on work design depend on its specific design features (Gagné et al., 2022), the generalizability of the results to other stationary, cognitive-assistive IAS needs to be investigated. Nevertheless, due to similar instructional materials for assembly processes, our results appear to be transferable, for example, to pick-to-light systems, augmented reality-based, and other projection-based IAS. Future studies should examine the role of specific design features and the generalizability of our study results.

### Conclusion

Our study contributes to the development of theories on the effect of innovative advanced technologies on work design by considering specific design features of an exemplary cognitive-assistive IAS in assembly (Gagné et al., 2022). We demonstrated the benefit of IAS for modern assembly workplaces in terms of enhanced feedback from the job and information processing. Particularly, our study emphasizes the benefit of IAS in employees’ daily work contrary to qualitative studies in which employees mentioned risks of restricted autonomy or systematic de-skilling when using IAS (Baethge-Kinsky, 2020; Blumberg & Kauffeld, 2020). Future studies should test if our results are transferable to other cognitive-assistive IAS.

## Supplementary Information

Below is the link to the electronic supplementary material.

Supplementary file1 (DOCX 25 KB)

Supplementary file2 (DOCX 908 KB)

## Data Availability

The authors pre-registered all procedures and hypotheses before data collection (https://osf.io/352zd/?view_only=5ff4cf3e692d44edbb0843418bb9bc26).

## Footnotes

1 Only in the German sample specialization showed rather low reliability (Cronbach’s α = .61).

2 A one-way MANCOVA with sample origin as a covariate revealed identical results, *F*(2, 192) = 5.530, *p* < .001, η_*p*_^2^ = .206, Wilk’s Λ = .794.
