# Supplementary material for: Investigating the effect of intelligent assistance systems on motivational work characteristics in assembly
Source: J Intell Manuf. 2023 Feb 22:1–14. Online ahead of print. doi: 10.1007/s10845-023-02086-4 (PMC9946279; doi:10.1007/s10845-023-02086-4)
Supplement: Supplementary file 1 — Supplementary file1 (DOCX 25 KB) [file 10845_2023_2086_MOESM1_ESM.docx]

**Table 1**

*Differences in demographic variables of German and English subsamples*

| Scale | German | | British | | Test | |  |
| --- | --- | --- | --- | --- | --- | --- | --- |
|  | M | SD | M | SD | |  | |
| Sex | 1.26 | 0.46 | 1.29 | 0.48 | | **χ²(2) = 0.334, *p* = .846, φ = .041** | |
| Age | 30.47 | 29.19 | 33.84 | 29.09 | | *t*(201) = -.826, *p* = .410 | |
| Prior experience with IAS | 1.89 | 0.31 | 1.84 | 0.37 | | **χ²(1) = 1.012, *p* = .314, φ = -.071** | |

Notes. IAS = Intelligent assistance system.

**Table 2**

*Distribution of the German and English subsample into the three experimental conditions*

| Condition | German subsample (*n* = 101) | English subsample (*n* = 102) | Total sample (*N* = 203) |
| --- | --- | --- | --- |
| Work without IAS | 31 | 35 | 66 |
| Work with IAS | 40 | 33 | 73 |
| Work with voluntary use of IAS | 30 | 34 | 64 |

*Notes.* IAS = Intelligent assistance system. A chi-sqaure test showed no significant differences in the distribution of the German and English subsamples into the three experimental conditions, χ²(1) = 1.159, p = .560, Cramér’s V = 0.076.

**Table 3**

*Internal consistencies (Cronbach’s* α) *of the study variables by German and English subsamples and total sample*

| Scale | | German subsample  (*n* = 101) | English subsample  (*n* = 102) | Total sample  (*N* = 203) |
| --- | --- | --- | --- | --- |
| 1 | Equipment use | .73 | .72 | .76 |
| 2 | Work scheduling autonomy | .79 | .91 | .86 |
| 3 | Decision-making autonomy | .86 | .89 | .89 |
| 4 | Work methods autonomy | .85 | .92 | .91 |
| 5 | Feedback from job | .88 | .83 | .86 |
| 6 | Job complexity | .87 | .85 | .86 |
| 7 | Problem solving | .88 | .80 | .84 |
| 8 | Informat. processing | .90 | .92 | .91 |
| 9 | Skill variety | .90 | .93 | .92 |
| 10 | Specialization | .61 | .89 | .79 |

*Notes*. The English version of the motivational work characteristics was assessed with the validated version of the Work Design Questionnaire (WDQ) (Morgeson & Humprhey, 2006). The German version was assessed by the validated German version of the WDQ (Stegmann et al., 2010).

**Table 4**

*Cell means and standard deviation of equipment use and motivational work characteristics according to experimental conditions*

|  | Work without IAS | | Work with IAS | | Work with voluntary use of IAS | |  |
| --- | --- | --- | --- | --- | --- | --- | --- |
|  | n = 66 | | n = 73 | | n = 64 | |  |
|  | M | SD | M | SD | M | SD | ANOVA |
| Work scheduling autonomy | 2.13 | 0.99 | 1.73 | 0.96 | 1.99 | 0.94 | *F*(2,200) = 3.109, *p* = .047, η^2^ = .030* |
| Decision making autonomy | 1.70 | 0.96 | 1.62 | 0.96 | 1.84 | 0.84 | *F*(2,200) = 1.048, *p* = .353, η^2^ = .010 |
| Work methods autonomy | 1.80 | 0.96 | 1.62 | 0.87 | 1.92 | 0.97 | *F*(2,200) = 1.829, *p* = .163, η^2^ = .018 |
| Feedback from job | 2.87 | 1.14 | 3.88 | 0.97 | 3.61 | 0.99 | *F*(2,200) = 17.483, *p* < .001, η^2^ = .149*** |
| Job complexity | 1.60 | 0.87 | 1.73 | 0.66 | 1.65 | 0.78 | *F*(2,200) = 0.482, *p* = .618, η^2^ = .005 |
| Problem solving | 1.55 | 0.69 | 1.65 | 0.83 | 1.59 | 0.61 | *F*(2,200) = 0.370, *p* = .691, η^2^ = .004 |
| Information processing | 1.75 | 0.91 | 2.13 | 1.02 | 1.99 | 0.85 | *F*(2,200) = 2.804, *p* = .063, η^2^ = .027 |
| Skill variety | 1.94 | 0.94 | 2.00 | 0.95 | 1.78 | 0.74 | *F*(2,200) = 1.115, *p* = .330, η^2^ = .011 |
| Specialization | 2.02 | 0.87 | 2.21 | 0.86 | 2.10 | 0.79 | *F*(2,200) = 0.857, *p* = .426, η^2^ = .008 |

*Notes*. IAS = Intelligent assistance system. * *p* < .05 *** *p* < .001.
