# Supplementary material for: Investigating the effect of intelligent assistance systems on motivational work characteristics in assembly
Source: J Intell Manuf. 2023 Feb 22:1–14. Online ahead of print. doi: 10.1007/s10845-023-02086-4 (PMC9946279; doi:10.1007/s10845-023-02086-4)
Supplement: Supplementary file 2 — Supplementary file2 (DOCX 908 KB) [file 10845_2023_2086_MOESM2_ESM.docx]

**Appendix A**

**Vignette of the condition work without IAS (translated from German)**

*Read the following job description carefully. Look at the fictitious workplace and try to put yourself in the situation.*

Imagine you are an employee in a medium-sized company, the Montage GmbH. You work in the assembly department and assemble boxes at the assembly workstations in the production halls of Montage GmbH.


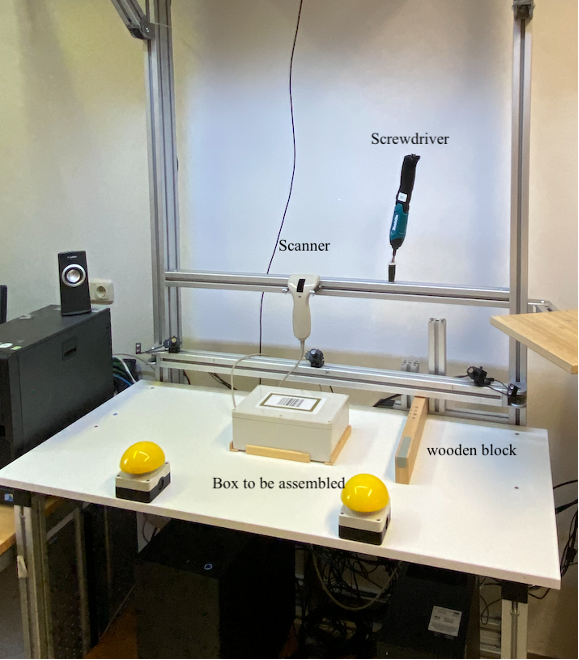


The picture shows an exemplary assembly workstation. On the work surface there is the box to be assembled and a wooden block with the required screws. The work is performed with a screwdriver and a scanner. After every 2 hours you assemble a different product.

**Appendix B**

**Vignette of the conditions work with IAS and work with voluntary use of IAS (translated from German)**


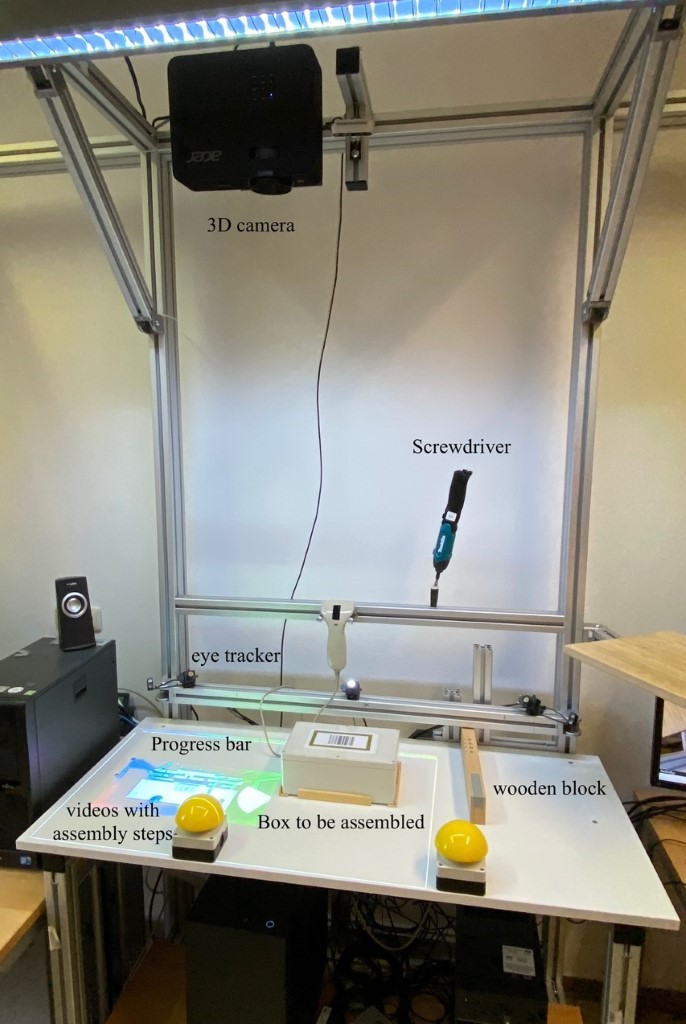
*Read the following job description carefully. Look at the fictitious workplace and try to put yourself in the situation.*

Imagine you are an employee in a medium-sized company, the Montage GmbH. You work in the assembly department and assemble boxes at the assembly workstations in the production halls of Montage GmbH.

The picture shows an exemplary assembly workstation. On the work surface there is the box to be assembled and a wooden block with the required screws. The work is performed with a screwdriver and a scanner. After every 2 hours you assemble a different product.

You will be supported by a digital assistance system while performing your job.

**It has the following features:**

- It guides you through each assembly step with the help of short videos, which are displayed on the work surface with the help of a beamer.
- For this purpose, your hand and eye movements are recorded using a 3D camera and three small, black eye-tracking cameras.
- If the assembly steps are performed correctly, the video lights up green on the work surface; if they are performed incorrectly, the video lights up red.
- The progress of your workflows is displayed in the form of a progress bar above the videos with the assembly steps on the work surface.
- Alternative workflows and assembly sequences can be automatically observed and taught using machine learning and artificial intelligence. This allows the system to constantly adapt to the user and other work steps, so that the system and the user can learn from each other.
